# The Southern Megalopolis: Using the Past to Predict the Future of Urban Sprawl in the Southeast U.S

**DOI:** 10.1371/journal.pone.0102261

**Published:** 2014-07-23

**Authors:** Adam J. Terando, Jennifer Costanza, Curtis Belyea, Robert R. Dunn, Alexa McKerrow, Jaime A. Collazo

**Affiliations:** 1 Southeast Climate Science Center, US Geological Survey, Raleigh, North Carolina, United States of America; 2 Department of Applied Ecology, North Carolina State University, Raleigh, North Carolina, United States of America; 3 Department of Biological Sciences, North Carolina State University, Raleigh, North Carolina, United States of America; 4 Core Science Analytics and Synthesis, US Geological Survey, Raleigh, North Carolina, United States of America; 5 U.S. Geological Survey, North Carolina Cooperative Fish and Wildlife Research Unit, North Carolina State University, Raleigh, North Carolina, United States of America; North Carolina State University, United States of America

## Abstract

The future health of ecosystems is arguably as dependent on urban sprawl as it is on human-caused climatic warming. Urban sprawl strongly impacts the urban ecosystems it creates and the natural and agro-ecosystems that it displaces and fragments. Here, we project urban sprawl changes for the next 50 years for the fast-growing Southeast U.S. Previous studies have focused on modeling population density, but the urban extent is arguably as important as population density per se in terms of its ecological and conservation impacts. We develop simulations using the SLEUTH urban growth model that complement population-driven models but focus on spatial pattern and extent. To better capture the reach of low-density suburban development, we extend the capabilities of SLEUTH by incorporating street-network information. Our simulations point to a future in which the extent of urbanization in the Southeast is projected to increase by 101% to 192%. Our results highlight areas where ecosystem fragmentation is likely, and serve as a benchmark to explore the challenging tradeoffs between ecosystem health, economic growth and cultural desires.

## Introduction

Cities are expanding, and as they do urban sprawl–low-density urban development outside the urban core–is expanding even more rapidly. In some regions, expansion of suburban habitats as a result of shifts to automobile-dependent living has led to increases in the urban footprint even where populations have not shown large increases [1]. Urban sprawl increases the connectivity among urban habitats while simultaneously fragmenting non-urban habitats such as forests and grasslands. These changes have a variety of effects on species and ecosystems, including impacts to water pollution, disturbance dynamics, local climate, and predator-prey relationships [2]–[5]. Urban sprawl will also, almost certainly, influence the ability of species to respond to climate change, in as much as it creates barriers to the movement of species that cannot survive in cities and corridors for those who can [6]. Knowledge about the potential future character of urban sprawl is thus useful to a variety of stakeholders, including resource managers, conservation organizations, and urban planners.

Any hope of integrating the effects of urbanization into management plans (whether for humans or wildlife), will depend on projections of urban sprawl. Such projections are typically generated using urban-growth models. The challenge is how to generate projections of urbanization that are robust enough to inform management priorities, decisions, and actions. In this regard, the challenge is similar to that faced when projecting climate change. In both cases, human actions taking place over decades will determine the outcome, and individual actions (global greenhouse gas emissions in the case of climate change; population growth, automobile dependency, and housing preferences in the case of urban growth) are difficult to predict on the time-scales of interest to decision-makers. In other words, the future as it relates to human actions has more uncertainty than what can be realistically quantified in an individual model.

A more cautious approach is to define scenarios that represent one or more particular kinds of futures, and then construct models to simulate the consequences of each scenario. For fast growing regions such as the Southeast US, the most relevant scenario for conservation and adaptation planning is the “business-as-usual” (BAU) scenario in which the net effect of growth is in line with that which has occurred in the past. While recent “Smart-Growth” initiatives that promote more intensive development and a return to a strong urban core are gaining popularity, this BAU scenario is still reflective of the primary development model. And without significant changes to the status quo, this type of growth will continue. Decision makers can use this information to see how the status quo, if continued, could affect and interact with the goals, objectives, and plans for the future.

Once the scenario is chosen, the urban-growth models typically use some combination of population density, land cover trends, and demographic models to set the parameters for the simulation. In this approach, the assumption is that changes in population lead directly to increased urbanization (by increasing density). This strategy has recently been used to project potential urban-induced threats to water quality for the U.S. and to project changes to forest stands in the Southeast [7]–[8]. But for many regions such as the Southeast that are heavily dependent on cars, the geographic extent of urbanization (which is dependent not only on population size but also road networks and the location of often far-flung industrial and commercial activity centers), may be as relevant to conservation and other management decisions as the density of people. And because sprawling, fragmented, or “leapfrog” development has been the dominant form of development in the Southeast [9], population growth models may under-predict the future extent of urban areas in this region.

Here we project urban growth to 2060 for the Southeast U.S. for a BAU scenario using a flexible cellular automata urban-growth model that focuses on changes in the extent of urban areas rather than the density of people within them. We use the SLEUTH model [10], which simulates patterns of urban expansion that are consistent with spatial observations of past urban growth and transportation networks. Natural and social land use controls, such as topographic barriers or regulatory restrictions in sensitive environmental areas are specified in the model parameterization and through resistance layers that reduce the likelihood of urbanization.

More sophisticated urban growth models exist that may include more complex parameterizations or explicit links to economic and demographic theory (e.g. [11]–[12]). However, these models typically can only be used over limited spatial extents because of intensive data requirements, often reaching to the individual parcel-level (e.g. [13]). We also note that while the simpler SLEUTH model has known issues due to its structure and assumptions for how urban growth occurs, any attempt to model the dynamics of systems and phenomena as complex as cities will require a significant level of abstraction. Because our aim is to produce projections at a fine spatial resolution over a multi-state area containing many dozens of cities, we used the SLEUTH model to take advantage of its scalability, its use of commonly available datasets, and the ability to focus on patterns of suburban and exurban development.

Our modeling approach has several advantages as it relates to projecting urbanization in this fast-growing region. The primary advantage is that street networks are used to define the urban extent, allowing for accurate mapping of suburban areas and enabling rapid updates to the model as conditions change. We also use a high spatial resolution for the projections (60 m) that better corresponds to typical suburban residential lot sizes than coarser scale models (e.g., 250 m), and reflects fine-scale impacts on habitat connectivity. We also use Monte Carlo simulation to better quantify the uncertainty in the model output. As modeled here, our projections reflect the most recent trends in the expansion of low-density urban areas. As such, they represent a BAU scenario depicting how urbanization may evolve in the Southeast U.S. given current policies, preferences and rates of growth. We analyze the results with respect to three questions of importance for conservation practitioners, land managers and urban planners:

•1) Given recent trends, what is the projected rate of urban growth for the next 50 years for this fast-growing region?
•2) Will this growth be uniform, or will some ecosystems and land cover types be more severely impacted than others?
•3) Which areas can be expected to become new growth centers?

## Methods

### Study Area

**Figure 1:**
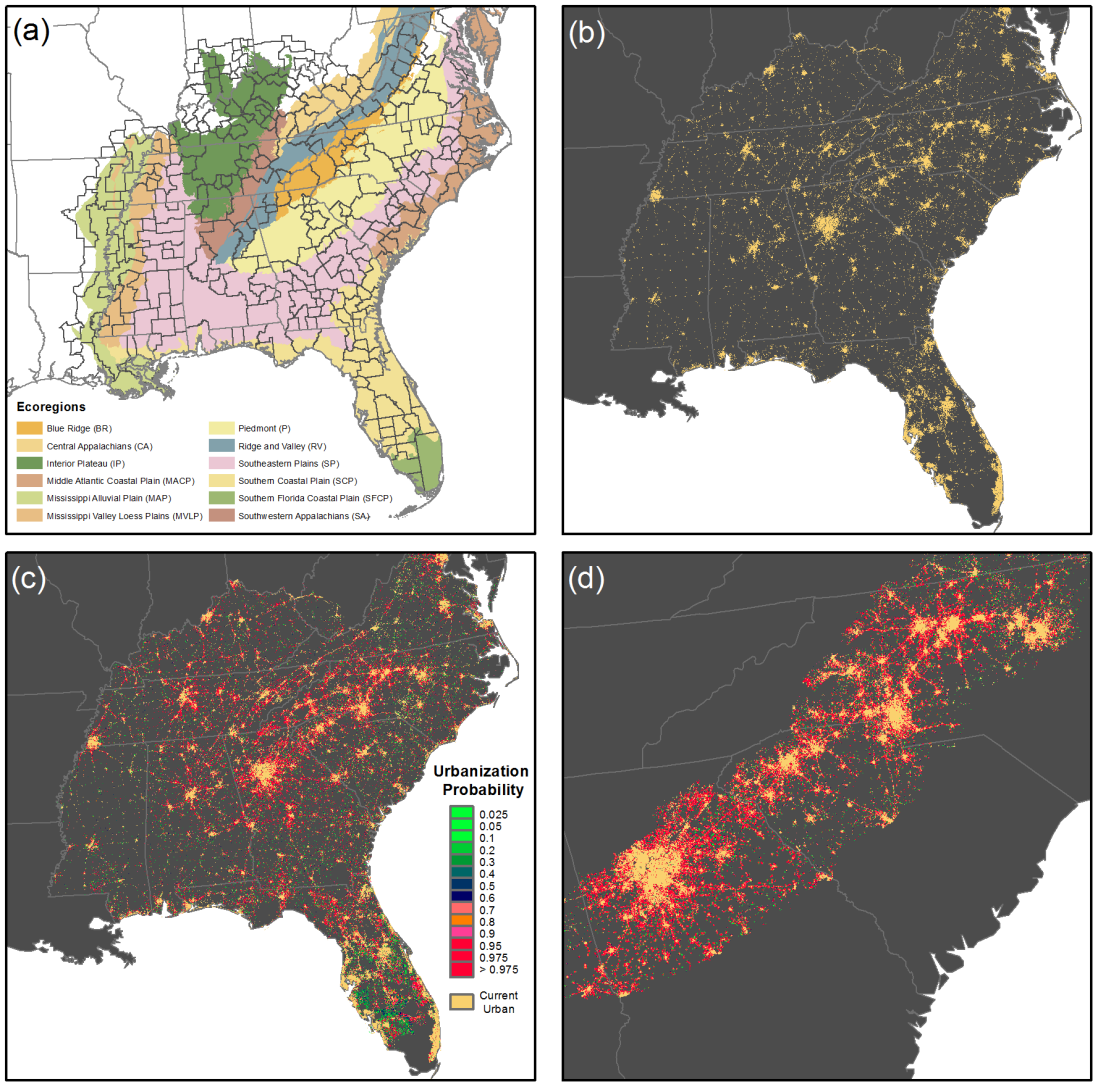
Business-as-usual urbanization scenario for the Southeast US. The Southeast US region used in this study. (a) EPA Level III ecoregions and initial urban extent. The 309 sub-regions (CSAs and rural county groups) used to calibrate the SLEUTH model are outlined in black. Red areas are urban extent as classified by our hybrid NLCD-TIGER dataset method (see File S1). (b) Initial urban land cover in 2009. (c) projected urban land cover in 2060. (d) projected urban land cover in Piedmont ecoregion, showing connected urban landscape.

We developed a baseline BAU urbanization scenario for a region in the Southeast U.S. that covers nine states (Figure 1). The Southeast has experienced explosive growth over the past 60 years, with a rate of population increase nearly 40% larger than the rest of the United States [14]. Over 77 million people now live in this region, where the typical new development pattern is suburban, automobile-dependent growth. This sprawling urbanization favors low-density development that requires large areas of land to support single-family housing and extensive road networks [15]. The region also contains high levels of plant and animal diversity, and many ecological communities in need of additional conservation [16]. For example, the once dominant but now endangered longleaf pine (*Pinus palustris*) ecosystem contains arguably the most species-rich communities outside of the tropics with many highly endangered species [17]. In addition, while climatic change in the Southeast is expected to be modest when compared to some other regions, the Southeast is at a high risk of the effects of sea level rise along its long, low-lying coast [18].

### Model Description and Data Layers

We used the SLEUTH urban-growth model [10], [19], a cellular automata model that simulates four types of urban growth patterns: spontaneous growth, new spreading urban centers, edge growth around existing urban areas, and road-influenced growth. Together these four growth types capture patterns of low to medium-density residential and commercial development (that is, extensive rather than intensive urbanization), which is consistent with the dominant urbanization patterns of the Southeast US (see File S1 for additional details). While noting the simplifying assumptions underpinning this approach (e.g. the model does not explicitly account for economic or demographic drivers of urban growth), the SLEUTH model has shown utility for planning purposes as it has evolved over the past 15 years and, encouragingly, showed skill in predicting future urban growth patterns even in its initial, simplest and coarsest iteration [20].

Four input data sets are required to calibrate the model growth parameters for simulation: (1) a layer indicating which areas are excluded from urban development or highly resistant to urbanization due to physical or regulatory constraints (e.g., water bodies, wetlands), (2) topographic data layers (which influence the ease of developing an area), (3) the transportation network for at least two time periods, and (4) the historic urban extent for at least three time periods. Inputs (1) – (3) were relatively straightforward to create across the Southeast (See File S1 for details of model inputs). Delineating the initial urban extent is critical to the calibration process but is a challenge for such a large region. We discuss our method for delineating recent urbanization patterns in the following section.

### Translating Road Networks into Proxies for Urban Growth

**Figure 2:**
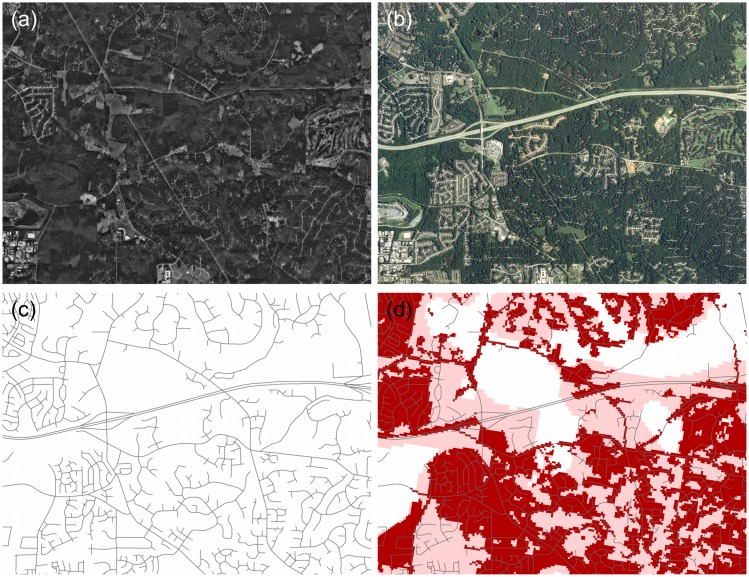
Urban sprawl examples and their representation in the input data. Imagery in (a) and (b) document the rapid but low-density urbanization common in the Southeast US (imagery for Raleigh, NC in 1993 and 2010). (c) Road network used as basis for identifying urban areas (same area as in (a) and (b)). (d) Initial (2009) urban classification (pink shade) based on road network density and NLCD urban classification (red shade).

We developed a process to classify past urbanized areas using data from the 2001 National Land Cover Dataset (NLCD) [21], and local street network information from the US Census Bureau’s Topographically Integrated Geographic Encoding and Referencing (TIGER) dataset [22]. Remotely sensed data (i.e. the NLCD) are an important part of our classification, however, we found that the urban classes in NLCD 2001 [21] tended to under-classify areas with low-density residential development– exactly the types of areas we are aiming to better characterize and project in this study. Therefore, we primarily relied on street network data as a proxy for developed areas. In doing so we assume that road networks that attain a certain density over time signal a transformation from un-developed and rural land parcels to developed and urban parcels (cf. [23]). We believe this assumption holds in the Southeast, where historically, new commercial or residential development coincides with an intensification and expansion of the existing road network. As the case of Raleigh, NC illustrates in Figure 2a and 2b, rapid expansion of suburban neighborhoods in the Southeast is characterized by extensive, fractal-like patterns of growth that accommodate cultural preferences for separate use zones (residential, commercial, industrial), which also creates distances that promote or even require automobile use to access goods and services.

There are several advantages in using road network data in the Southeast US as a proxy for urban areas. One advantage is that the data are updated regularly (typically every one to three years) for each state and territory in the US. This frequent updating ensures that rapid changes in development patterns, such as housing booms or recessions, can be captured in the calibration process. It also allows for more robust model fitting over time since more observations become available over a shorter time period. Second, streets and roads are, in general, consistently categorized even though each state is responsible for providing the data to the federal government as part of the US Census TIGER program. Finally, residential areas may be more accurately represented by digital street networks than remotely-sensed imagery, particularly in forested areas, which is commonly the case in the Southeast US.

To classify urban areas we used TIGER data collected for four years (2000, 2006, 2008, and 2009) to delineate the street network and the changing extent of urbanized areas. Earlier years were available, but were of poor quality. Inaccurate delineation of the road network was still present in many areas in the first year. In such areas, the next available year with accurate road delineation was substituted for the year 2000 data. We note that this process should not significantly bias model calibration since the more recent line-work depicted roads that were already present in the first year.

We then calculated network density values and retained locations with street densities that were consistent with urban areas (see File S1 for further details). We intersected these locations with NLCD classified urban pixels. Finally, we added locations that crossed another, higher street density threshold. This enabled the inclusion of areas that are not classified as urban in the NLCD land cover but nonetheless are more suburban or exurban in character (see Figure 2c and 2d for an illustration of the classification, and File S1, Figure S1 and Table S1 for accuracy assessment results).

### Model Projections

We used the resulting historic urban extent to simulate urban growth for 309 aggregated county sub-regions (Figure 1). The sub-regions closely adhered to the U.S. Census Bureau’s Core Based Statistical Areas which consist of aggregations of economically and demographically linked counties. The resulting sub-regions should reflect more or less consistent internal growth rates, which limits low growth areas across the Southeast from having undue influence on the projected urbanization in high growth areas in other parts of the Southeast, and vice versa.. The simulation covers the time period 2010–2060. We ran 200 independent Monte Carlo simulations for each sub-region to quantify the uncertainty in our projection. The Monte Carlo simulations result in some model runs that depict more aggressive urbanization compared to others, while still being consistent with recent urban growth rates.

## Results

### Urbanization Projections

**Figure 3:**
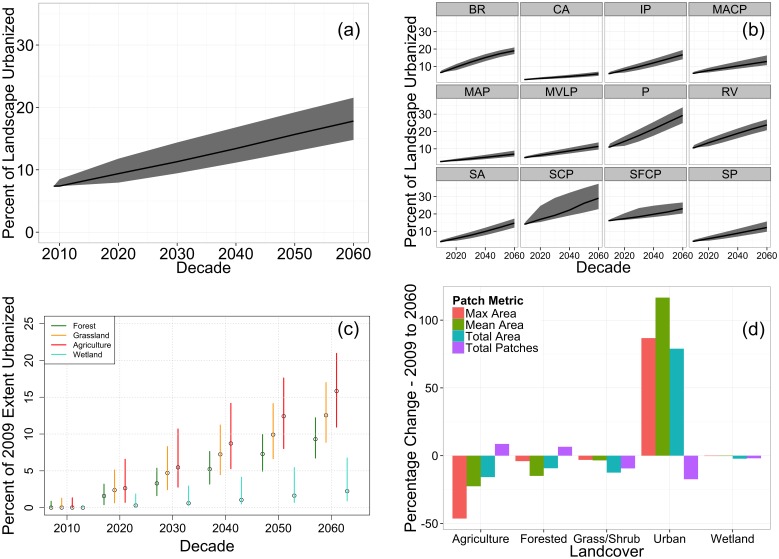
Land cover change metrics. (a) and (b) show time series of projected urbanization for 200 model simulations for the study region and twelve ecoregions, respectively. (c) The 95% projected range of the proportion of each land cover type converted to urban. (d) Change in patch metrics for all land cover types between 2009 and 2060. Ecoregion abbreviations in (b) are as follows: BR – Blue Ridge, CA – Central Appalachians, IP – Interior Plateau, MACP – Mid Atlantic Coastal Plain, MAP – Mississippi Alluvial Plain, MVLP – Miss. Valley Loess Plains, P – Piedmont, RV – Ridge and Valley, SP – Southeastern Plains, SCP – Southern Coastal Plains, SFC – South Florida Coastal Plain, SA – Southwest Appalachian.

In our projections, the urban footprint will greatly increase over the next 50 years (Figure 1). The median projection shows that the amount of land in urban areas increases by 139%, from 90,700 km^2^ (7.4% of land area) in 2009 to 216,900 km^2^ (17.8%) in 2060 (Figure 3a). The expansion is not uniform across the region. The largest absolute change is in the Piedmont ecoregion, which includes many of the largest metropolitan centers in the Southeast, such as Atlanta and Charlotte. In this region, urban areas expanded 165%, from 17,800 km^2^ in 2009 to 47,500 km^2^ in 2060. The largest proportional increase is in the Southwestern Appalachian ecoregion, where urban areas are projected to expand by 261%, from 1,500 km^2^ in 2009 to 5500 km^2^ in 2060 (Figure 3b). In contrast, the smallest modeled proportional increase (42%) is in the Southern Florida Coastal Plain ecoregion.

**Figure 4:**
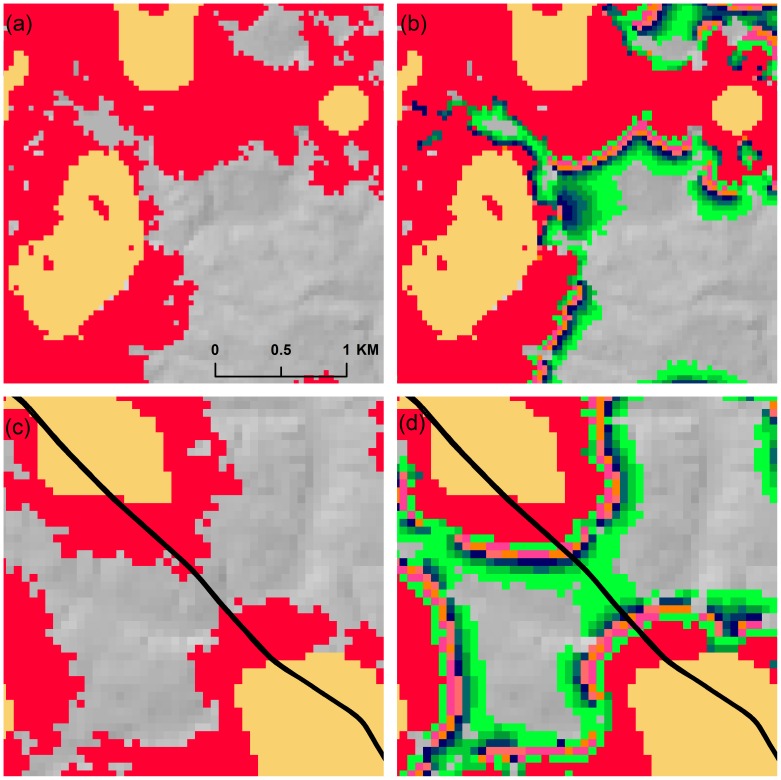
Examples of SLEUTH model output. Individual fifty-year model simulations (2010–2060) along with the final projection based on 200 Monte Carlo simulations for two fast-growing regions: Walton County in Georgia (Figure 4a and 4b) and Wake County in North Carolina (Figure 4c and 4d). Red cells in (a) and (c) correspond to new urban growth and gold cells depict 2009 classified urban areas. Cell colors in (b) and (d) are the same as color legend in Figure 1.

While most Monte Carlo simulations show similar patterns of growth, interesting variations are apparent at the local level (Figure 4). In some cases, that growth can result in the connection of two urban centers. In Figure 4a, extensive new growth areas resulting from a single Monte Carlo simulation are visible (shown in red). However, Figure 4b indicates that other Monte Carlo simulations resulted in even more aggressive growth patterns, seen here as the large area of blue-green cells in the center of the image. Figure 4c is an example of possible urbanization along road corridors, which in this case results in a small chance (<10% or <20 Monte Carlo simulations) that these two spreading suburban areas will become connected (Figure 4d).

Comparisons of model projections across land cover types show large differences in urban growth (Figure 3c). The largest conversion is from agriculture to urban land use, in which the 95% range of projected losses (i.e. percentage of all agriculture lands that are converted to urban) is 11% - 21% by 2060. For grassland, the projected loss is 9% - 17%, followed by projected forest losses of 7% - 12%. Wetland losses are much lower owing to the higher resistance to urbanization specified in the model (see File S1). As a result, only 1% - 7% of wetlands are projected to be converted to urban areas. However in two mountainous ecoregions, Blue Ridge and Central Appalachians, the lack of developable land led to much higher conversion rates from wetland to urban land cover (between 5% - 33% of Blue Ridge wetlands and 3% - 13% of Central Appalachians wetlands converted to urban areas, respectively).

We summarized the effect of urbanization on landscape fragmentation in the Southeast by overlaying model output on a generalized version of the 2006 National Land Cover Database (NLCD). We then calculated four simple patch metrics for each land cover class: total area of each land cover type, mean patch area, maximum patch size, and total number of patches. Urban areas become more connected, with fewer, larger patches in 2060 compared to 2009. On average in our simulations, urban patch size increases in area by 79%. This is in contrast to agriculture and forest land cover types, which become more fragmented with smaller patch sizes (Figure 3d). Agricultural land uses experience the greatest fragmentation, with a mean 46% decline in the largest patch size and a 22% decline in mean patch size. Wetland areas showed the least change because of their high resistance to urbanization in the model.

## Discussion

Our results point to a future where urban areas occupy a much greater portion of the landscape of the Southeast U.S. The projected region-wide increase in urban area would constitute a doubling or tripling of land devoted to urban and suburban uses. With this increase will come greater need for urban infrastructure, but also an increase in all of those ecological features associated with urbanization including urban run-off, urban warming and habitat fragmentation.

The tremendous growth in urbanization will come at the expense of natural areas as well as agricultural and silvicultural landscapes. Furthermore, the growth will be uneven and focused in areas that have few geographic and socioeconomic constraints, or in areas with high aesthetic value that act as strong attractants for development. As such, the largest urban expansions are projected in Blue Ridge, Ridge and Valley, Southern Coastal Plain, and Piedmont ecoregions. We also project new urban centers in the Appalachian Mountains and central Florida, while the more aggressive model simulations show large new areas of urbanization north of the Everglades region. The greatest expansion, projected to occur in the Piedmont ecoregion, reflects a combination of growth attractors such as the existence of large urban areas, a lack of geographic constraints on growth, auto-oriented residential development, and proximity to natural amenities (Appalachian Mountains and the Atlantic Ocean). The rapid urbanization projected to occur in the mountainous regions also results in greater wetland losses because topographic constraints and the abundance of protected forest areas limit the supply of alternative land use types.

Undoubtedly our model simulations do not capture the full range of uncertainty, and our focus on a single BAU scenario does not consider alternative policies that could promote different urbanization patterns. However, the broad patterns of growth do reflect the recent trends, both in terms of the speed at which urbanization has progressed in the Southeast, and in the locations that are most affected by it. Other studies operating on similar temporal and spatial scales have shown lower rates of urbanization [7], [24]. One possible reason is that our more aggressive urbanization scenario is not constrained by population projections that previously have underestimated this region’s population growth [25]. Furthermore, our model calibration covers a period of very rapid expansion of suburban development (along with the beginning period of the global recession that prompted a similarly rapid retreat from building new housing). In effect this is a true Business As Usual scenario, albeit one that likely portrays an upper bound benchmark for urban growth, which visualizes the consequences of continuing current land use policies and implicitly reflects factors that have contributed to rapid development in this region (e.g., favorable climate and a cultural tendency toward sprawling growth).

We are projecting changes in the spatial footprint of urban areas, and in doing so, do not model the differences in the types of urbanization within those urban areas. Not all areas classified as “urban” are alike: cities are heterogeneous in terms of their land use, population density, and impacts [26]–[27]. Still, our focus on the spreading frontier of development underscores the increasing connectedness and favorable conditions for urban-adapted species, while a whole host of species and ecosystems will experience reduced habitat area and increased difficulty in migration and dispersal.

The changes we project would have significant and lasting effects on the region’s ecosystems. The increasingly fragmented natural landscape would reduce habitat availability, suppress natural disturbance processes (such as wildfires), hinder management actions that come into conflict with urban areas, and likely eliminate existing corridors. Furthermore, all these impacts could occur simultaneously, posing a particularly devastating threat to already vulnerable species and systems. Such is the case for the endangered Red-cockaded Woodpecker (*Picoides borealis*) in the longleaf pine (*Pinus palustris*) ecosystem, where planned stepping-stone corridors are expected to be negatively impacted by encroaching urbanization, and will likely make management of existing fire-suppressed habitat difficult [28], [3].

At the same time, urban corridors will expand and become less fragmented which will promote the establishment of novel habitats that favor an entirely different assemblage of species (e.g., [29]–[30]). For example our results show the emergence of a new, completely connected megalopolis in the Piedmont region by 2060, extending from Raleigh, NC to Atlanta, GA (Figure 1d). Not only will habitats and corridors for wildlife be eliminated, but the continuous urban corridor will have a warmer climate than surrounding rural areas. Urban heat islands in this region are 0.5°–1.5°C warmer than rural areas [31], meaning that the new megalopolis would effectively extend the warmer southern and coastal climates to the (formerly cooler) Piedmont, which could be 2°–6°C warmer due to climate change [32]. Others have shown that these urban and suburban habitats are already acting as corridors for the expansion of invasive species that take advantage of urban heat island conditions [4]; a phenomenon likely to accelerate as these urban corridors expand in a warming climate.

Projections of the future have, for good reasons, tended to focus on global warming. However, global warming scenarios will be superimposed on or even act synergistically with urbanization scenarios. In the Southeast US, the effects of global warming are expected to be modest compared with many regions, however our results suggest that the effects of urbanization, given business-as-usual will not be. Given that urbanization has many consequences for how both humans and other species live, optimizing such growth could become a key national and regional priority, where optimization includes providing for biodiversity as well as economic development and cultural desires. However, history suggests humans, in contrast to ants and slime molds (e.g. [33]), rarely optimize growth, particularly when multiple objectives such as profit, equity, and ecological integrity come into conflict. Given this reality, and the not unlikely possibility that the recent urbanization path will continue, our model suggests the template around which natural resource managers, urban planners and everyone else whose job relates to the distribution of wild places or humans, will need to respond.

## Supporting Information

Figure S1 **Stem-plots of commission and omission error percentages for 32 sampled CSAs.** The two stem-plots in the left column are results for sampled urban pixels in the CSAs and the two-stem plots in the right column are results for sampled rural pixels. Color-coded numbers indicate the number of points out of the 272 randomly sampled points in each CSA that were classified as urban or rural during manual photo interpretation.
(TIFF)

Table S1 **Pooled accuracy assessment results for 32 sampled CSAs.**
(DOCX)

File S1 **Detailed Description of Model Calibration and Accuracy Assessment.**
(DOCX)
