# Supplementary figures and images for: The Southern Megalopolis: Using the Past to Predict the Future of Urban Sprawl in the Southeast U.S
Source: PLoS One. 2014 Jul 23;9(7):e102261. doi: 10.1371/journal.pone.0102261 (PMC4108351; doi:10.1371/journal.pone.0102261)

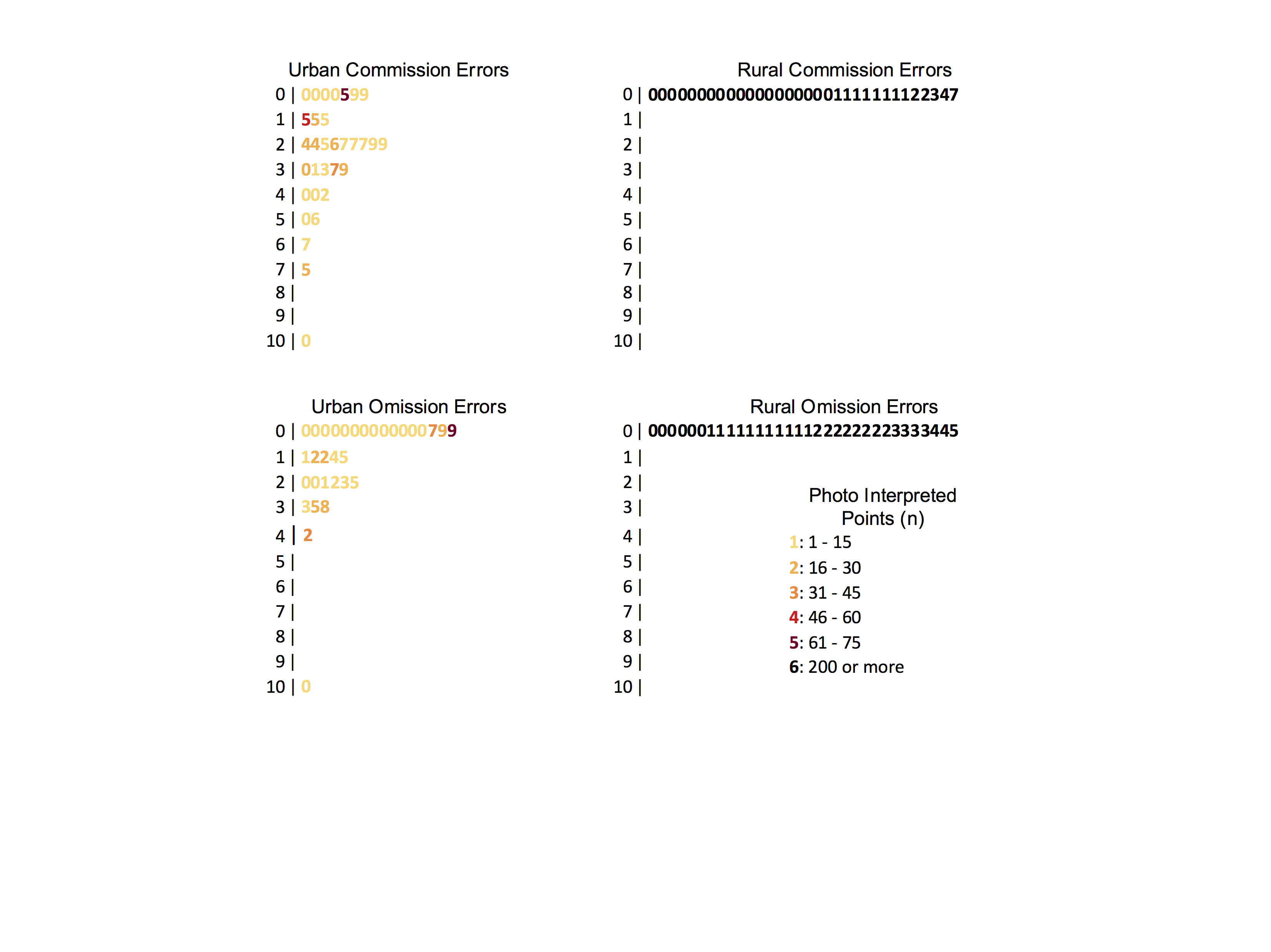

Supplement: Figure S1 — Stem-plots of commission and omission error percentages for 32 sampled CSAs. The two stem-plots in the left column are results for sampled urban pixels in the CSAs and the two-stem plots in the right column are results for sampled rural pixels. Color-coded numbers indicate the number of points out of the 272 randomly sampled points in each CSA that were classified as urban or rural during manual photo interpretation. (TIFF) [file pone.0102261.s001.tiff]
