# Supplementary material for: The Southern Megalopolis: Using the Past to Predict the Future of Urban Sprawl in the Southeast U.S
Source: PLoS One. 2014 Jul 23;9(7):e102261. doi: 10.1371/journal.pone.0102261 (PMC4108351; doi:10.1371/journal.pone.0102261)
Supplement: Table S1 — Pooled accuracy assessment results for 32 sampled CSAs. (DOCX) [file pone.0102261.s002.docx]

| All CSAs | Model Classification | |  | Omission | Commission | Overall Mapping |
| --- | --- | --- | --- | --- | --- | --- |
| Photo Classification | Urban | No Urban | Total Possible | Error | Error | Accuracy |
| Urban | 373 | 69 | 442 | 16% | 26% | 98% |
| No Urban | 129 | 8133 | 8262 | 2% | 1% |  |
| Total | 502 | 8202 | 8704 |  |  |  |

Table S1: Pooled accuracy assessment results for 32 sampled CSAs.
