# Supplementary material for: The Southern Megalopolis: Using the Past to Predict the Future of Urban Sprawl in the Southeast U.S
Source: PLoS One. 2014 Jul 23;9(7):e102261. doi: 10.1371/journal.pone.0102261 (PMC4108351; doi:10.1371/journal.pone.0102261)
Supplement: File S1 — Detailed Description of Model Calibration and Accuracy Assessment. (DOCX) [file pone.0102261.s003.docx]

**SUPPORTING INFORMATION FILE S1**

**The Southern Megalopolis: Using the past to predict the future of urban sprawl in the Southeast U.S. – Terando AJ et al.**

**DETAILED DESCRIPTION OF MODEL CALIBRATION AND ACCURACY ASSESSMENT**

*Model Description and Data Layers*

The SLEUTH urban-growth model [1,2] requires four different types of spatial data: (1) a layer indicating which areas are excluded from urban development or highly resistant to urbanization (such as water bodies, protected habitat, or wetlands), (2) the local slope gradient which indicates topographic constraints to urbanization, (3) the transportation network for at least two time periods (usually defined as streets and roads), and (4) historic urban extent for at least three time periods. These data layers are used to calibrate five parameters or growth coefficients (known as Dispersion, Breed, Slope, and Road Gravity) that vary between 0 and 100 after calibration and determine the expansion rate and pattern of urban growth in the model (see Table I for descriptions of these parameters). The exclusion data layer is derived from the 2001 National Land Cover Dataset (NLCD; [3]) and the Protected Areas Database of the US (PADUS; <http://gapanalysis.usgs.gov/padus/>). The exclusion layer also varies between 0 and 100 and acts as a resistance to urbanization in the model where higher values result in increasingly lower probabilities of urbanization, independent of the predicted likelihood of that location's becoming urbanized according to the five growth parameters. We fix the exclusion layer probabilities at 1 (equivalent to a model value of 100) for protected areas and 0.95 for wetlands (i.e. high resistance to urbanization). Slope data are derived from the National Elevation Dataset (NED; <http://ned.usgs.gov/>), while transportation data are obtained from the U.S. Census Bureau TIGER Line Dataset [4].

*Translating Road Networks into Proxies for Suburban Growth*

Several prior studies using SLEUTH for local applications have mapped the observed urban extent using aerial photos or historic maps (e.g. [5,6]). This strategy was not feasible for this study given the need for a consistent classification of urban and suburban areas across a large spatial extent. An alternative is to use remotely sensed imagery that is classified into land cover classes (such as NLCD developed land cover classes), or imagery that serve as proxies of urbanization (e.g., impervious surface, cf. [2]). However there are limitations to this approach as well. For example, in the case of impervious surface cover, our initial tests that used these data as a surrogate for urban extent showed unacceptably high mis-classification of suburban and exurban areas that are likely due to higher rates of tree canopy cover. More broadly, while the NLCD urban classes and other derived remotely sensed urban land cover products such as the North American Landscape Characterization (NALC; http://www.epa.gov/esd/land-sci/north-am.htm) can provide useful approximations of suburban development, they are updated infrequently (typically on the order of five to ten years for NLCD), have large time periods in between imagery (e.g., one image each decade for three decades for NALC), or use different techniques to characterize development [7], which increases the difficulty of comparing patterns across time periods.

To classify urban areas we began with the first historic urban time period (2000) and selected areas classified as one of four NLCD urban land cover classes for the 2001 imagery (Developed Open Space, and Low, Medium, or High Intensity Development Classes). We intersected these grid cells with a layer representing street density where individual grid cells had values greater than 33 m/10,000 m^2^ in a one square kilometer area. We then included all cells in the street density layer in the first time period that had densities greater than 50 m/10,000 m^2^. This allowed us to include areas that are not classified as urban in the NLCD land cover but nonetheless are more suburban or exurban in character, as exemplified by the denser residential street networks. These threshold values were settled on after experimenting with a variety of thresholds in the Raleigh-Durham, NC metropolitan region. The results of our accuracy assessment (discussed in the following section) confirmed that these thresholds successfully captured most urbanized areas in the study region. By taking the spatial intersection of these two datasets, we constructed an urban layer based on two independent sets of data, one of which is updated frequently with a consistent methodology since 2000. For this study, the most recent NLCD land cover was not yet available to incorporate into our historic urban extent. Therefore, differences in the three subsequent urban layers (for years 2006, 2008, and 2009) are solely due to the addition of new grid cells that have the higher road density threshold.

*Capturing Sub-regional Patterns of Development*

Because we are simulating urban growth patterns over such a large area, it is necessary to sub-divide the region for computational tractability, but also to capture different rates and patterns of urbanization that result from differing rates of population growth, economic activity, land use policies, and environmental constraints. Accordingly we created sub-regions based on the U.S. Office of Management and Budget (OMB) Combined Statistical Areas (CSAs; [8]). CSAs are aggregations of individual counties that are associated with each other because of shared commuting patterns that reflect economic and social ties. As such they are a good proxy for regions that share similar development patterns. To account for rural counties that are not part of a CSA, we combined counties into sub-regions if they were contiguous and were within the same state. If a rural county was contiguous to another rural county, but they were in different states, they were split into separate sub-regions based on the assumption that different states may have controlling regulations that impact development patterns. Conversely, if a county is part of a metropolitan CSA but is not in the same state as the central metropolitan county, we still included that county in the sub-region for analysis. A total of 309 sub-regions and CSAs were delineated through this process (see Figure 1 in main text).

*Model Calibration and Evaluation*

Model calibration involves an iterative search through combinations of the five growth coefficients to select the best fit between the simulated and observed urban patterns. Because SLEUTH is a cellular automata model based on location-specific urbanization probabilities, each time a simulation is run with one set of growth parameters the resulting urban patterns will be slightly different. Therefore, for the calibration process we ran 25 simulations for each parameter combination in each sub-region. The combination of large parameter space (equal to 1 X 10^10^ possible combinations), the size of the analysis region consisting of 309 sub-regions, and the additional simulations required to better evaluate the model fit results in a very high computational burden. As such, we took several steps to reduce to size of the parameter space so that it would still be feasible to carry out the calibration process.

The first step was to fix the road gravity coefficient at 100, allowing for roads to have maximum influence on urbanization. This choice was based on findings by [9] that the road gravity coefficient did not clearly impact the model fit, and therefore the overall model performance, thus holding it at a fixed value should not materially affect the results. We also reduced the computation costs of the parameter search by fixing the slope coefficient at 25 in coastal plain ecoregions. Here we made use of the fact that there is very little topographic variation in this physiographic region, which suggests slope is not likely to constrain urbanization. In other ecoregions the slope parameter was calibrated along with the other coefficients. There is also a critical slope threshold above which urbanization cannot occur in the model. The default threshold is 21% and we increased this threshold in high topographic relief areas where significant amounts of urbanization occurred.

For the remaining possible parameter combinations, we calculated the percent error between model values and observations using three spatial fit metrics: total number of urbanized pixels (i.e. total urban area), the number of urban edge pixels, and the number of urban clusters which represent contiguous urban areas. We limited the choice of parameter combinations to those with a maximum error of ±5% for the total number of urbanized pixels. The idea being that the total urban area was the most important criteria for the model to accurately predict. An overall error score was calculated by normalizing the fit metrics to the error values that resulted from setting all growth coefficients to 100 and then summing the normalized error values. This parameter combination produces a very high error score since it allows for runaway, unchecked urban growth. Thus it represents a reasonable standard to use as the "worst case" for model calibration, against which all subsequent parameter combinations can be measured. The parameter combination with the resulting lowest relative error score was used to simulate future patterns of urban growth for each sub-region.

*Accuracy Assessment*

We performed an accuracy assessment to evaluate the efficacy of our method for characterizing urbanized areas. Thirty-two of the 309 sub-regions were randomly selected for the assessment. The sub-regions were chosen according to a gamma distribution (with parameters empirically derived from all sub-regions), to ensure that rare but important high-population areas would be included in the analysis. Within each sub-region we randomly sampled 272 locations for comparison, yielding an expected 5% accuracy error at the 0.9 confidence level and assuming no prior knowledge of the probability of correctly classifying the location as urban or rural [10]. Sampled locations were classified as either urban or rural using imagery from Google Earth^TM^ for the closest date to 2009, the final year in the calibration phase of the model.

We show the pooled error estimates in Table S1, with errors of omission and commission. As expected when classifying a relatively rare land class (urban) compared to a common class (rural), the misclassification rates are roughly an order of magnitude higher for the urban classification, but still low overall, with a commission error rate of 26% and an omission error rate of 16%. This is in contrast to commission and omission error rates of 1% and 2%, respectively for the rural classification.

The variance of the misclassification errors amongst sub-regions is also much higher for the urban locations compared to the rural locations (Figure S1). This can be seen in the color-coded stem plots in Figure S1, where all rural locations had low errors of commission and omission (less than 10%), and were also sampled at high rates (200 or more of the 272 sampled locations, shown as bolded black numbers). Conversely, the areas that were classified as urban (the two stem-plots in the left-hand column of Figure S1) had a wide range of error rates, from 0 to 100% error. However as denoted by the color-coded numbers, the sub-regions that had more urban locations among the 272 sampled locations (which corresponds to high population areas) also had lower misclassification rates compared to the urban pixels sampled in low-population rural regions. Thus, the presence of some high misclassification rates is not likely to bias the region-wide urbanization simulations because these regions are predominantly rural with few urban areas to serve as growth catalysts.

*Patch Metrics*

Summary patch metric statistics were calculated for each land cover type for the initial period (2009) and the final year of the simulation (2060). Land cover was derived from the 2001 NLCD. Patch metrics calculated included: total area of each land cover type, largest patch size (ha), mean patch size, and number of patches. Patches were delineated using the “Region Group” command in ArcGIS™.

**References:**

1. Clarke KC, Gaydos LJ (1998) Loose-coupling a cellular automaton model and GIS: long-term urban growth prediction for San Francisco and Washington/Baltimore. Int J Geogr Inf Sci 12: 699–714.

2. Jantz CA, Goetz SJ, Donato D, Claggett P (2010) Designing and implementing a regional urban modeling system using the SLEUTH cellular urban model. Comput Environ Urban Syst 34: 1–16.

3. Homer C, Dewitz J, Fry J, Coan M, Hossain N, et al. (2007) Completion of the 2001 National Land Cover Database for the Conterminous United States. Photogramm Eng Remote Sens 73: 337–341.

4. US Census Bureau (2007) TIGER Products. 2006 Second Ed TIGER/Line Files. Available: http://www.census.gov/geo/maps-data/data/tiger.html. Accessed 24 June 2014.

5. Herold M, Goldstein NC, Clarke KC (2003) The spatiotemporal form of urban growth: measurement, analysis and modeling. Remote Sens Environ 86: 286–302.

6. Silva EA, Clarke KC (2005) Complexity, emergence and cellular urban models: lessons learned from applying SLEUTH to two Portuguese metropolitan areas. Eur Plan Stud 13: 93–115.

7. Vogelmann J, Howard S, Yang L, Larson C, Wylie B, et al. (2001) Completion of the 1990s National Land Cover Data set for the conterminous United States from Landsat Thematic Mapper data and Ancillary data sources. Photogramm Eng Remote Sensing 67: 650–662.

8. US Office of Management and Budget (2010) 2010 standards for delineating metropolitan and micropolitan statistical areas. Federal Register 75: 37246-39052.

9. Jantz CA, Goetz S, Shelley M (2004) Using the SLEUTH urban growth model to simulate the impacts of future policy scenarios on urban land use in the Baltimore-Washington metropolitan area. Environ Plan B-PLANNING Des 31: 251–271.

10. Meidinger D (2003) Protocol for accuracy assessment of ecosystem maps. Research Branch, B.C. Ministry of Forests, Victoria, B.C. Technical Report 011.
